# Robust Electrospinning-Constructed Cellulose Acetate@Anthocyanin Ultrafine Fibers: Synthesis, Characterization, and Controlled Release Properties

**DOI:** 10.3390/polym14194036

**Published:** 2022-09-27

**Authors:** Mingzhu Liu, Shilei Zhang, Yuanyuan Ye, Xiaoqing Liu, Jiangling He, Lingfeng Wei, Die Zhang, Jiaojiao Zhou, Jie Cai

**Affiliations:** 1 National R&D Center for Se-Rich Agricultural Products Processing, Hubei Engineering Research Center for Deep Processing of Green Se-Rich Agricultural Products, School of Modern Industry for Selenium Science and Engineering, Wuhan Polytechnic University, Wuhan 430023, China; 2 Key Laboratory for Deep Processing of Major Grain and Oil, Ministry of Education, Hubei Key Laboratory for Processing and Transformation of Agricultural Products, Wuhan Polytechnic University, Wuhan 430023, China

**Keywords:** cellulose acetate, cellulose acetate@anthocyanin ultrafine fiber, robust electrospinning, stability, controlled release

## Abstract

Anthocyanin has attracted increasing attention due to its superior biological activity. However, the inherently poor stability of anthocyanin limits its practical applications. In this study, a fast and straightforward method was developed to improve the stability of anthocyanin. Cellulose acetate ultrafine fiber-loaded anthocyanin (CA@Anthocyanin UFs) was prepared by robust electrospinning, and the potential application of cellulose acetate ultrafine fibers (CA UFs) as a bioactive substance delivery system was comprehensively investigated. The experimental results showed that CA@Anthocyanin UFs had protective effects on anthocyanin against temperature, light, and pH. The results of the artificially simulated gastric fluid (pH = 2.0) indicated that the CA@Anthocyanin UFs had a controllable release influence on anthocyanin. A 2,2-Diphenyl-1-picrylhydrazyl (DPPH) radical-scavenging assay suggested that the CA@Anthocyanin UFs still had an excellent antioxidant activity similar to anthocyanin. This work demonstrated the potential application of robust electrospinning-constructed cellulose acetate ultrafine fibers in bioactive substance delivery and controlled release systems, as well as its prospects in green packaging due to the nature of this environmentally friendly composite.

## 1. Introduction

Anthocyanin, as a class of water-soluble natural food pigments, is rich in resources, safe, and non-toxic. It is widely found in fruits, vegetables, grains, and other plants, giving plants different colors such as blue, red, or purple [1]. Anthocyanin is a kind of flavonoid compound formed by the binding of anthocyanidin with various sugars through glycosidic bonds. Anthocyanin is classified according to the types of sugars bonded with anthocyanidin [2]. Anthocyanin belongs to the polyphenol family of compounds and is also an important bioactive substance with various functions and nutritional values [3]. It is widely used for scavenging pure radicals [4], cancer treatment [5], tumor ablation [6], its anti-inflammatory aspects [7], protecting eyesight, losing weight, and preventing diabetes [8]. With the development and deepening of its research, various effects of anthocyanin have been revealed, and it has been found that anthocyanin has great utilization values and application prospects in food [9], medicine [10], cosmetics [11], and other fields [12]. However, anthocyanin is extremely unstable. During storage and processing, its stability can be easily affected by external environmental conditions, such as temperature, light, pH, oxygen, and metal ions, which can reduce its biological activity, resulting in a low bioavailability and limiting its applications [1]. Therefore, it is necessary to explore ways to improve the stability of anthocyanin and expand the applications of anthocyanin in various fields.

A large number of studies have reported that anthocyanin can be loaded into various delivery systems, including biopolymer-based nanoparticles, nanogels, and complex coacervates, to improve its stability and biological activity [13]. These delivery and controlled release systems not only protect the biological activity of anthocyanin, but they can also control its release characteristics. Therefore, the selection of the correct materials is crucial to improving the stability and bioavailability of anthocyanin. Electrospinning is a simple and effective method used to prepare continuous fibers based on polymers and composites, and it has become a research hotspot in recent years [14,15,16]. Electrospun nanofibers have the advantages of a large surface area, small fiber diameter, high porosity, and other unique abilities, which can improve drug-loading efficiency, reduce the sudden release of a drug, and ensure the safety of a drug’s application [17]. They have shown great potential in drug and bioactive substances’ delivery and release. Ahmad et al. developed a new type of rod-shaped implantable drug delivery systems (IDDS) by electrospinning cellulose acetate and polycaprolactone nanofiber membranes [18]. The results showed that the developed nanofiber membranes were suitable for long-term drug delivery via their implantation in subcutaneous tissues. Han et al. prepared lutein-loaded polyvinyl alcohol/sodium alginate nanofibers by electrospinning and further evaluated their release behavior, which confirmed the potential of polyvinyl alcohol/sodium alginate electrospinning-constructed fibers for bioactive substance delivery and controllable release [19].

Cellulose acetate (CA) is a good candidate for use in bioactive substances’ delivery and controlled release among the different types of materials. It is a kind of regenerated cellulose fiber obtained by the esterification reaction of cellulose and acetic acid, possessing the characteristics conducive to environmental protection, security, and good degradation [20]. Thus, CA is widely used as a drug-carrying fiber to load a variety of drugs and small molecules [21]. CA-electrospun nanofibers loaded with gallic acid showed controllable release characteristics, antioxidant activity, and antibacterial activity, whereby CA-electrospun fibers could serve as gallic acid carriers in a transdermal drug delivery system and wound-dressing scenario [22]. Yang et al. prepared clear core–shell nanofibers through a modified triaxial–electrospinning process—a mixture of ibuprofen and gliadin fibers with a thin layer of CA in the nanocores. A dissolution test in vitro showed that the existence of the CA coating eliminated the initial burst release of ibuprofen from the drug–protein complex, thereby prolonging the release time, which was proportional to the coating thickness. The research provided a new method for developing novel functional nanomaterials [23]. Milovanovic et al. employed CA as a carrier of thymol and explored the effect of the thymol content on CA, thymol’s release kinetics, and antibacterial activity. The results have also shown that CA, as a controlled release carrier of thymol, has wide application prospects [24].

Empowered by the preparation of nanofibers by the electrostatic-spinning method, CA can greatly embed various biologically active substances and has the characteristics of environmental protection, naturality, non/low toxicity, and good degradation. Herein, CA ultrafine fibers loaded with anthocyanin (CA@Anthocyanin UFs) were prepared via a robust electrospinning process, and the microstructure and thermal stability of the samples were further analyzed. In addition, the stability, controlled release performance, and oxidation resistance of the composite film were evaluated. The information obtained in this study can provide new ideas and methods for the development of nanomaterials used for loading and delivering bioactive substances. In addition, this work also offers potential prospects in green packaging.

## 2. Materials and Methods

### 2.1. Materials

Cellulose acetate (CA, Mn ~30,000) was provided by Sigma-Aldrich (St. Louis, MO, USA). Acetone was purchased from Hengfa Chemical Reagent Co., Ltd. (Tianjin, China). Methanol anhydrous, anhydrous citric acid, sodium citrate, sodium chloride, sodium hydroxide, N, N-Dimethylacetamide (DMAc), and 2,2-Diphenyl-1-picrylhydrazyl (DPPH) were obtained from Sinopharm Chemical Reagent Co., Ltd. (Shanghai, China). Anhydrous ethanol was provided by Kermel Chemical Reagent Co., Ltd. (Tianjin, China). Hydrochloric acid was purchased from China Pingmei Shenma Group Kaifeng Dongda Chemical Co., Ltd. (Kaifeng, China).

### 2.2. Synthesis of CA@Anthocyanin UFs

Cellulose acetate@anthocyanin ultrafine fibers (CA@Anthocyanin UFs) were fabricated using a needle-based, temperature-assisted electrospinning setup. Details of the fabrication of CA@anthocyanin UFs are as follows. A total of 1.44 g of CA was dissolved in 9 mL of DMAc, acetone, and anhydrous methanol homogeneous solution (1:1:1, *v*/*v*/*v*) under constant magnetic stirring at room temperature for 1.5–2 h to obtain completely dissolved CA solution (16%, *w*/*v*). Various mass fractions (3 wt.%, 5 wt.%, 7 wt.%, 9 wt.%, and 11 wt.%) of anthocyanin were then added into the CA solution by constant stirring at room temperature until the anthocyanin was dissolved to obtain the electrospinning solution.

Then, the electrospinning solution was placed in a syringe and pumped by a syringe pump at a flow rate of 1.0 mL/h. The voltage power was set at 20 kV and the distance from the needle tip to the collector was 16 cm, according to the previous electrospinning process but with some changes [25]. The nanofibers were collected in a cylinder collector wrapped with conductive aluminum foil. Electrospinning was conducted at room temperature throughout the experiment. Finally, CA@Anthocyanin UFs were obtained, and pure cellulose acetate ultrafine fibers (CA UFs) without anthocyanin were prepared under the same conditions for comparative analysis.

### 2.3. Characterizations

Morphological characterizations of the surfaces of cellulose acetate ultrafine fiber– loaded anthocyanin (CA@Anthocyanin UFs) and neat cellulose acetate ultrafine fibers without anthocyanin (CA UFs) were carried out using a scanning electron microscope (SEM, Hitachi S-3000N, Tokyo, Japan). The average diameters were obtained based on SEM images by using an image analysis software (ImageJ 1.51). Fourier transform infrared (FTIR) spectra were determined by a NEXUS670 spectrometer (Thermo Nicolet Corporation, Madison, WI, USA) and the scanning wavelength range was 4000–400 cm^−1^. A thermogravimetric analysis (TGA) was performed on a TGA/DSC/1100SF instrument (Mettler Toledo Instruments Co., Ltd., Shanghai, China) in an N_2_ atmosphere with a temperature range of 30–800 °C and a heating rate of 10 °C/min. Differential scanning calorimetry (DSC) analysis was carried out on a DSC instrument (TA Instruments, Netzsch, Germany) in an N_2_ atmosphere at a temperature range of 25–300 °C and a heating rate of 10 °C/min. X-ray diffraction (XRD) patterns were recorded on a X-ray diffractometer (PANalytical Empyrean, Almelo, The Netherlands) with Cu-Kα radiation. Dynamic mechanical analysis (DMA) was performed on a DMA Q800 instrument (TA Instruments, New Castle, DE, USA) at a speed of 2 mm/min. The absorbance was performed using the WFJ7200 visible spectrophotometer (Unico Instrument Co., Ltd., Shanghai, China).

### 2.4. The Degree of Swelling of CA UFs and CA@Anthocyanin UFs

The swelling degree of ultrafine fibers is responsible for its controlled release properties. Electrospun ultrafine fibers with identical sizes (20 × 20 mm^2^) were immersed in 1.0 M of phosphate buffer saline solutions at a stirring rate of 50 rpm for 24 h. The degree of swelling of ultrafine fibers was calculated using the following equation [26]:

Degree of swelling (%) = (W_2_ − W_1_)/W_1_ × 100 (1)

where W_1_ and W_2_ represent the weight of dried ultrafine fibers and swollen ultrafine fibers, respectively.

### 2.5. Determination of Anthocyanin Loading Efficiency

A total of 0.2 g of CA@Anthocyanin UFs were shredded and immersed in 10 mL of acetone; then, 200 mL of distilled water was added, under continuous magnetic stirring. Then, the sample solution was evaporated until the volume was about 10 mL with a rotary evaporator at 37 °C, and the solution was filtered and collected. The absorbance value was measured; then, the amount of anthocyanin in the solution was calculated according to the standard curve (Figure S1). The above steps were repeated in the precipitate, and finally, the amount of anthocyanin in 0.2 g of CA@Anthocyanin UFs was calculated by the following formulas [27]:

M_1_ = W_1_ × V_1_ M = (M_1_ + M_2_ + M_3_ + … + Mn)/0.2 Loading efficiency (%) = (M/N) × 100(2)

where W_1_, V_1_, M_1_, and N represent the anthocyanin amount in solution according to the standard curve, volume of solution after rotary evaporation, the anthocyanin amount of the filtrate, and the original anthocyanin mass fraction of CA@Anthocyanin UFs, respectively.

### 2.6. Effects of Temperature, Natural Light, and pH on the Stability of Anthocyanin and CA@Anthocyanin UFs

In order to explore the effect of temperature on the stability of pure anthocyanin, 50 mg of pure anthocyanin was weighed, deposited into a test tube with a stopper, and placed in the dark for 2 h and 6 h in a water bath at 30, 60, and 90 °C. Then, the samples were immediately cooled in an ice bath and diluted to 100 mL with distilled water. The loss rate was calculated by measuring the absorbance value of the sample solution. To research the effect of temperature on CA@Anthocyanin UFs, 0.2 g of CA@Anthocyanin UFs was weighed and put into a cuvette with a stopper, placed in a water bath at 30, 60, and 90 °C in the dark for 2 h and 6 h, and then cooled in an ice bath immediately. A total of 10 mL of acetone was added to completely dissolve the CA@Anthocyanin UFs, and then 200 mL of distilled water was added. Then, the volume of the mixture was evaporated to below 10 mL using a rotary evaporator, followed by the introduction of water to raise the volume by 10 mL for the calculation of loss rate.

In order to explore the effect of natural light on the stability of pure anthocyanin and CA@Anthocyanin UFs, a certain amount of pure anthocyanin was weighed and stored in a petri dish under room lights. A total of 50 mg of pure anthocyanin was extracted every 2, 4, 6, and 8 days, and water was added to a constant volume of 100 mL to measure the absorbance value and calculate the loss rate. Similarly, a certain amount of the CA@Anthocyanin UF sample was weighed and placed in a petri dish under room light. A total of 0.2 g of CA@Anthocyanin UFs was extracted every 2, 4, 6, and 8 days and completely dissolved with 10 mL of acetone. A total of 200 mL of distilled water was added and then evaporated to below 10 mL using a rotary evaporator. Then, water was added to a constant volume of 10 mL, followed by filtration treatment. Finally, the absorbance value was recorded, and the loss rate was calculated.

In order to explore the effect of pH on the stability of pure anthocyanin and CA@Anthocyanin UFs, a citric acid/sodium citrate buffer solution was prepared with pH = 2.0, 4.0, 6.0, 7.0, 8.0, and 10.0. A certain amount of anthocyanin and CA@Anthocyanin UF sample was placed in a colorless transparent vial containing the above buffer solution and left in the dark. The color changes of anthocyanin and CA@Anthocyanin UFs were observed at 0 h, 1 h, 2 h, 4 h, and 8 h.

### 2.7. Determination of Controlled Release Properties of CA@Anthocyanin UFs In Vitro

The release characteristics of anthocyanin were studied by artificially simulating gastric fluid in vitro. A total of 0.3 g of CA@Anthocyanin UF (7 wt.%) was added into 20 mL of artificially simulated gastric fluid (pH = 2.0), which was placed in a vibrator under a temperature of 37 °C and a speed of 50 r/min. One sample (from a total of eight samples) was taken every 30 min and filtered in the dark. Anthocyanin content and release rate were calculated by measuring the absorbance [18].

Release rate (%) = m_1_/(m × 7 wt.% × n) × 100 (3)

where m_1_, m, and n are the amount of released anthocyanin, the amount of CA@Anthocyanin UFs, and the loading efficiency of CA@Anthocyanin UFs (7 wt.%), respectively.

### 2.8. Antioxidant Activity of Anthocyanin and CA@Anthocyanin UFs

The antioxidant activity of CA@Anthocyanin UFs was illustrated by measuring the DPPH radical-scavenging capability of the released anthocyanin from CA@Anthocyanin UFs, modified from a previously reported method [4]. A total of 7.88 mg of DPPH was dissolved in 100 mL of absolute ethanol to prepare 0.2 mmol/L DPPH solution. A total of 4 mg of anthocyanin and 115.78 mg of CA@Anthocyanin UFs were added into 100 mL of acetone and methanol anhydrous homogeneous solution (1:1 = *v/v*), respectively, to obtain test specimens. Afterwards, 2 mL of test solution was mixed with 2 mL of DPPH solution. Then, the solution was shaken for 1 min and reacted for 30 min at room temperature in the dark. The absorbance of the solution at a wavelength of 517 nm (A_1_) was measured using a UV–Vis spectrophotometer. At the same time, DPPH ethanol solution was replaced with an equal volume of anhydrous ethanol, and the absorbance value was denoted as A_2_. The sample solution was replaced with an equal volume of anhydrous ethanol, and other operations followed the same procedure. The absorbance value was determined as A_0_. The modified equation to determine the antioxidant activity of both the anthocyanin and the CA@Anthocyanin UFs is as follows [4]:

DPPH radical-scavenging rate (%) = [1 − (A1 − A2)/A_0_] × 100 (4)

The measurements were conducted in triplicate. By comparing the DPPH radical-scavenging rates of anthocyanin and CA@Anthocyanin UFs, the standard deviation was calculated, and the significance was analyzed.

### 2.9. Statistical Analysis

The statistical analyses of the data were performed by Duncan’s test using SPSS-26. Different letters represent significant differences (*p* < 0.05) between groups.

## 3. Results and Discussion

### 3.1. The Morphology and Loading Efficiency of CA@Anthocyanin UFs

**Scheme 1:**
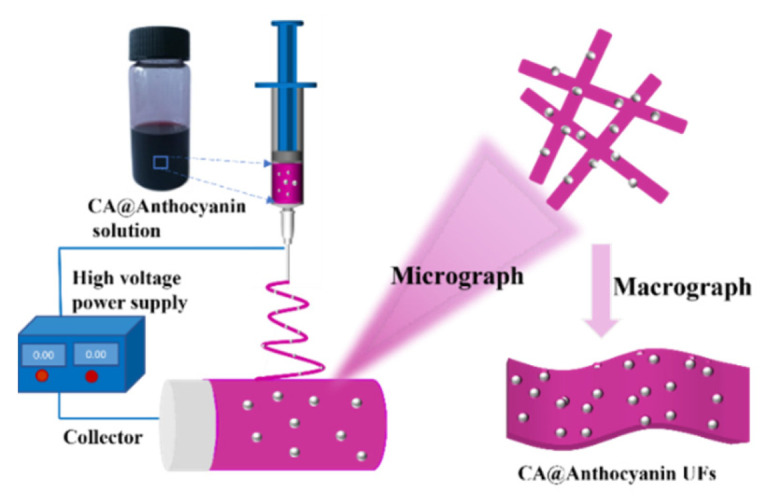
Schematic illustration of as-prepared CA@Anthocyanin UFs by electrospinning.

The process followed for preparing the cellulose acetate@anthocyanin ultrafine fibers (CA@Anthocyanin UFs) by robust electrospinning is shown in Scheme 1.

**Figure 1:**
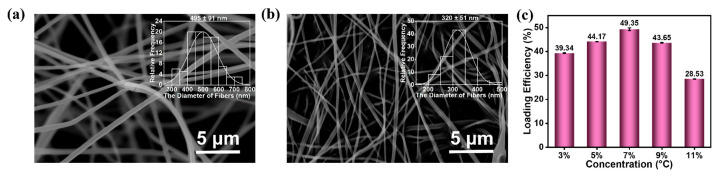
(**a**,**b**) SEM images of the CA@Anthocyanin UFs with various concentrations of anthocyanin (inset: diameter distribution of CA@Anthocyanin UFs; (**a**)―0%; (**b**)―7%); (**c**) loading efficiency of the CA@Anthocyanin UFs with various concentrations of anthocyanin.

The relationship between the absorbance and anthocyanin content was measured by determining the standard curve of the anthocyanin aqueous solution. The loading efficiency and scanning electron microscope (SEM) images of the CA@Anthocyanin UFs carrying anthocyanin at different concentrations are shown in Figure 1 and Figure S2. As demonstrated, with the increasing anthocyanin content in the spinning solution, the loading efficiency of anthocyanin first increased and then decreased gradually. The CA@Anthocyanin UFs carrying 7 wt.% anthocyanin had a better loading efficiency (49.35%). Much higher concentrations of anthocyanin can affect the properties of the spinning solution, and the spinning fluid is prone to drop to hinder the normal spinning process. It can also be seen that a higher concentration of anthocyanin has more obvious effects on the nanofibrous morphology. As shown in Figure 1a, the pure CA UFs were smooth. With the gradual addition of anthocyanin into the spinning solution, the diameter of the electrostatic spinning fiber was decreased. This was because the addition of anthocyanin reduced the viscosity of the spinning solution and made the electrospinning jet easy to stretch, which led to the decreased fiber diameter [28]. When the content of anthocyanin was 7 wt.%, the fiber diameter was small and relatively uniform, smooth (without beads), and microfibers with a good morphology were formed. When the anthocyanin content exceeded 7 wt.%, the fiber diameter became inconsistent, which was caused by the excessive anthocyanin concentration. This resulted in too much surface tension, and the high-voltage electrostatic field did not completely overcome the surface tension of the spinning solution (Figure S2). Therefore, the CA@Anthocyanin UFs (7 wt.%, Figure S3) with the highest anthocyanin loading were selected for the subsequent experimental studies.

### 3.2. FTIR of Pure Anthocyanin, CA UFs, and CA@Anthocyanin UFs

**Figure 2:**
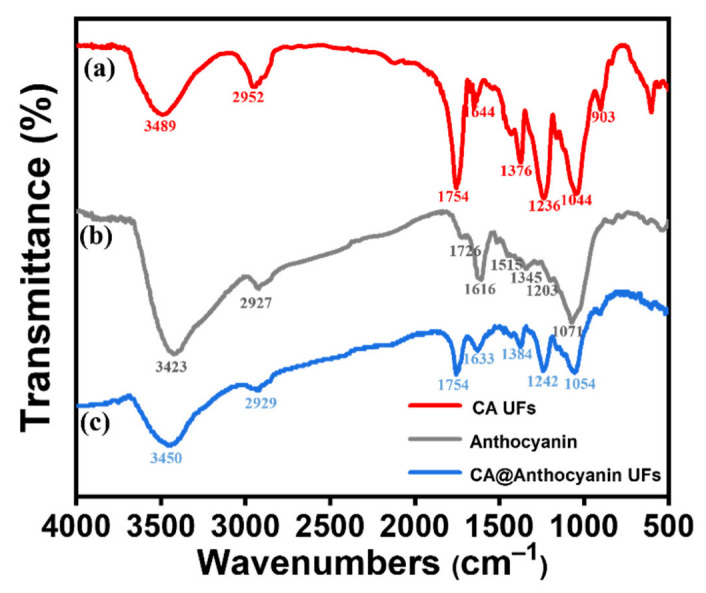
FTIR spectra of (a) CA UFs, (b) anthocyanin, and (c) CA@Anthocyanin UFs.

The Fourier transform infrared (FTIR) spectra of the CA UFs, pure anthocyanin, and CA@An thocyanin UFs are shown in Figure 2. According to Figure 2a, the stretching vibration peak of the hydroxyl group at ~3489 cm^−1^, the stretching vibration peak of C=O at ~1754 cm^−1^, the stretching vibration peak of C-O-C at ~1236 cm^−1^, and the stretching vibration peak of C-O at ~1044 cm^−1^ are characteristic peaks of CA. The FT-IR spectra of anthocyanin showed characteristic peaks at ~3423 cm^−1^ (O-H stretching vibration), ~1616 cm^−1^ (aromatic ring stretching vibration), and ~1071 cm^−1^ (C-H bending vibration) (Figure 2b). It was worth noting that after the addition of anthocyanin, the CA@Anthocyanin UFs (Figure 2c) covered typical peaks of CA and anthocyanin, and the position of the characteristic peak of the CA UFs was not significantly changed. Therefore, both CA and anthocyanin were successfully coupled into the UFs.

### 3.3. Thermal Properties of CA UFs and CA@Anthocyanin UFs

**Figure 3:**
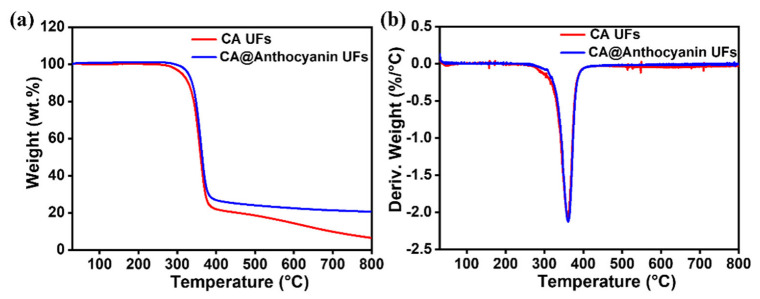
(**a**) TGA and (**b**) DTG curves of the CA UFs and CA@Anthocyanin UFs under N_2_ atmosphere.

The thermogravimetric analysis (TGA) and derivative thermogravimetry (DTG) curves intuitively show the changes in the thermal properties of the nanocomposites (Figure 3 and Figure S4). There was only one obvious period of rapid decline at ~300 °C. After adding a certain amount of anthocyanin into the CA system, the degradational trend of the CA@Anthocyanin UFs was similar to that of the CA UFs.

The CA UFs and CA@Anthocyanin UFs were stabilized up to ~250 °C and then began to degrade. After that, they stabilized at ~390 °C. The CA UFs were degraded stably until reaching 10 wt.% residue. However, the CA@Anthocyanin UFs were degraded stably, with a residue of 20 wt.%. The experimental results were consistent with a previous study [29]. The CA UFs and CA@Anthocyanin UFs had a nearly vertical thermogravimetric process between 300–400 °C [30], indicating that the sample itself was undergoing a violent decomposition reaction, which caused an about 75 wt.% mass loss. This also showed that the CA UFs and CA@Anthocyanin UFs were relatively stable below 300 °C.

Differential scanning calorimetry (DSC) was also used to evaluate the thermal stability of the CA UFs and CA@Anthocyanin UFs (Figure S5). The CA UFs showed a significant endothermic peak at ~228 °C, which was slightly higher than the melting temperature (T_m_) of CA powder (~207 °C) [31]. The CA@Anthocyanin UFs showed an endothermic peak at ~227 °C. This indicated that the addition of 7 wt.% anthocyanin could not significantly influence the T_m_ of the CA UFs, aside from a slight decrease in T_g_ from 199.54 °C to 198.87 °C, which was observed for the CA UFs after the introduction of anthocyanin. Hence, the introduction of anthocyanin has a quite slight effect on the T_m_ and T_g_.

### 3.4. Crystallinity and Mechanical Properties of CA UFs and CA@Anthocyanin UFs

It can be seen from Figure S6 that the X-ray diffraction (XRD) pattern of anthocyanin showed a broad peak near 2*θ* = 20°, which indicated that the anthocyanin was amorphous [32]. The diffraction peak intensity of the CA UFs decreased after anthocyanin was introduced. Hence, the addition of anthocyanin led to the reduced crystallinity of the CA UFs.

The mechanical properties of the CA UFs and CA@Anthocyanin UFs are shown in Figure S7. The tensile strength of the CA@Anthocyanin UFs was lower than that of the CA UFs, which was due to the addition of anthocyanin that could reduce the compactness of the CA UFs [33]. In addition, the elongation at break of the CA@Anthocyanin UFs decreased slightly compared with the CA UFs, which resulted from the fact that anthocyanin could hinder the interactions between fibrous chain–chain interactions within CA, reducing the flexibility of the CA@Anthocyanin UFs [34].

### 3.5. Swelling Properties of CA UFs and CA@Anthocyanin UFs

**Figure 4:**
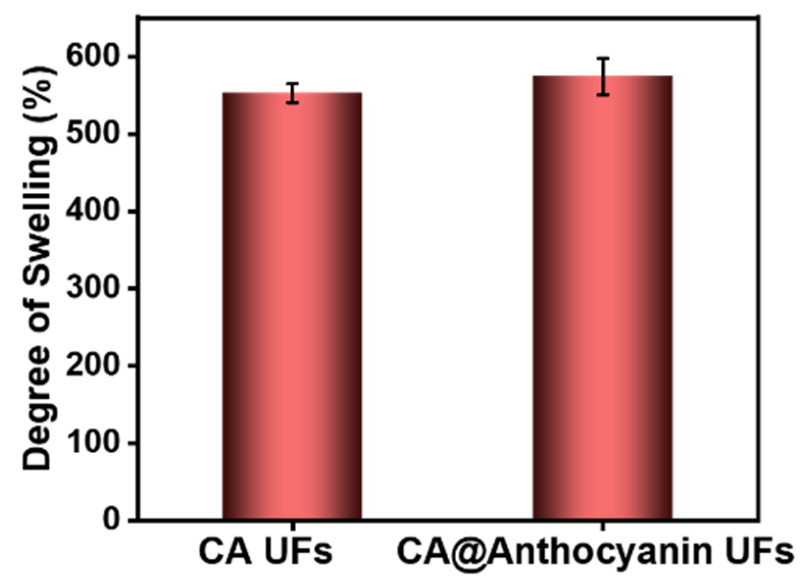
The degree of swelling of the neat CA UFs and the CA@Anthocyanin UFs.

As shown in Figure 4, the swelling degree of the CA UFs was ~553%, and that of the CA@Anthocyanin UFs displayed a higher swelling degree of ~574%. This result was in agreement with the previously reported works [35,36], where anthocyanin has the excellent hydrophilic nature.

### 3.6. Effects of Temperature on the Stability of Anthocyanin and CA@Anthocyanin UFs

**Figure 5:**
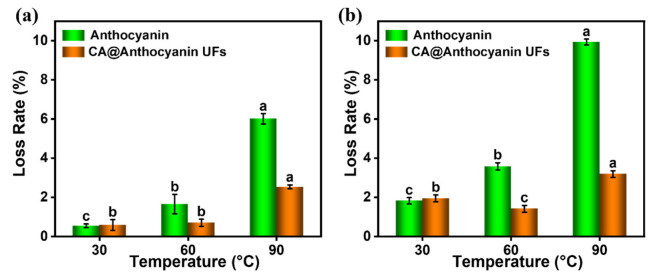
Loss rate of the anthocyanin and CA@Anthocyanin UFs under various water bath temperatures: (**a**) water bath time—2 h; (**b**) water bath time—6 h. Note: Different letters (a, b, c) represent significant differences (*p* < 0.05) between groups.

The loss rate of the anthocyanin at different temperatures is shown in Figure 5a (2 h) and Figure 5b (6 h). The loss rate of pure anthocyanin and anthocyanin loaded in CA@Anthocyanin UFs increased with the increase in the water bath temperature. The loss rate of anthocyanin and the CA@Anlthocyanin UFs changed less at lower temperatures. However, the loss rate of pure anthocyanin increased more significantly when the temperature was higher.

The loss rate of anthocyanin increased from 6.01% to 9.93% after 2 h and 6 h in the 90 °C water bath, while the loss rate of the CA@Anthocyanin UFs only increased from 2.54% to 3.19%. The CA@Anthocyanin UFs showed a significantly lower loss rate than pure anthocyanin at 90 °C. This was probably because anthocyanin was quite sensitive to heat and unstable at high temperatures; therefore, the increase in temperature led to the rupture of glycoside bonds and promote anthocyanin degradation [37]. This phenomenon was consistent with the result of a previous study, which reported that a high temperature led to the degradation of anthocyanin and that the degradation rate of anthocyanin was proportional to temperature under certain conditions [38]. Wojdyło et al. also reported that the degradation of anthocyanin was related to temperature and that the anthocyanin in the products stored at low temperature was more stable [39]. The loss rate of pure anthocyanin was higher than that of the CA@Anthocyanin UFs (Figure 5). Therefore, the CA@Anthocyanin UFs could greatly protect anthocyanin from degradation.

### 3.7. Effects of Natural Light on the Stability of Anthocyanin and CA@Anthocyanin UFs

**Figure 6:**
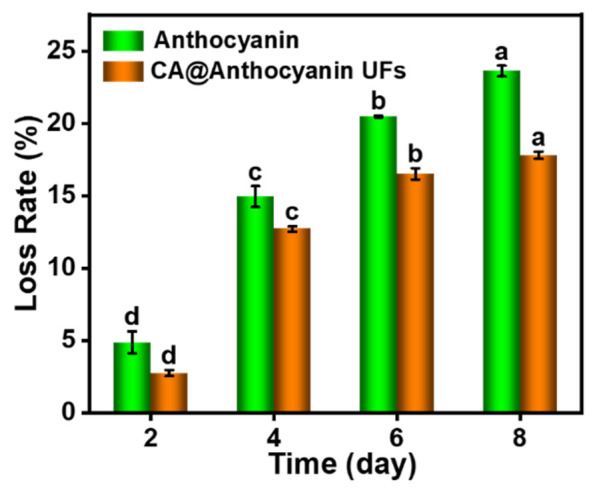
Loss rate of the anthocyanin and CA@Anthocyanin UFs under various natural light days. Note: Different letters (a, b, c, d) represent significant differences (*p* < 0.05) between groups.

Under light irradiation, the acyl group on the molecular structure of anthocyanin was prone to detachment and might have caused other degradational reactions to reduce the stability of anthocyanin. Natural light contains many ultraviolet rays, which also lead to the oxidation or decomposition of anthocyanin and reduce its stability [40]. As shown in Figure 6, the loss rate of anthocyanin and the CA@Anthocyanin UFs increased with the increasing natural light irradiation time. This phenomenon occurred because natural light can also promote the degradation of anthocyanin since there are enough ultraviolet rays within natural light.

However, the loss rate of anthocyanin in the CA@Anthocyanin UFs was always smaller than that of pure anthocyanin. After 8 days of illumination, the loss rate of anthocyanin in the CA@Anthocyanin UFs was only 17.79%, while the loss rate of pure anthocyanin was 23.62%, which is 1.33 times that of the CA@Anthocyanin UFs. Herein, the degradation rate of anthocyanin loaded in cellulose acetate was lower than that of pure anthocyanin. Therefore, the CA@Anthocyanin UFs had a certain protective effect on anthocyanin, which could inhibit the degradation of anthocyanin from natural light and protect the light stability of anthocyanin.

### 3.8. Effects of pH on the Stability of Anthocyanin and CA@Anthocyanin UFs

pH affects the structure of anthocyanin; consequently, the stability of the anthocyanin in this study was affected [1]. In the solution with different pH levels, the surface structure of anthocyanin was altered, leading to a color difference. Anthocyanin was mainly present as a stable red to orange colored AH^+^ form at pH ≤ 2.0. When the pH = 3.0–6.0, anthocyanin existed predominantly in the form of a red to blue mixture of neutral tautomeric Q^−^ bases. The anionic base Q^−^ was formed via increasing the pH (pH > 8.0). Under alkaline conditions, the structure of pure anthocyanin was changed, the color was obviously deepened, and the stability and its activities were gradually decreased with the increasing the pH values [41].

**Figure 7:**
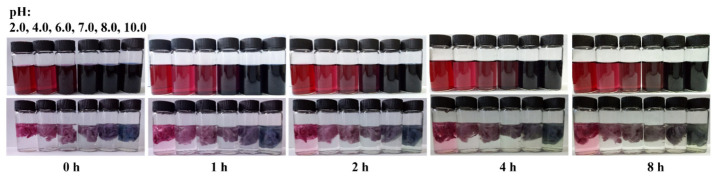
Physical appearance of the anthocyanin (**top**) and CA@Anthocyanin UFs (**bottom**) with various pH conditions (pH = 2.0, 4.0, 6.0, 7.0, 8.0, and 10.0) and various standing times (t = 0, 1, 2, 4, and 8 h).

In Figure 7, the color of the anthocyanin solution gradually changes from red to dark blue with the increase in pH (2.0–10.0). Compared with the pure anthocyanin, a weaker color change of the CA@Anthocyanin UFs was observed, which demonstrated that the CA@Anthocyanin UFs had some protective effect on anthocyanin against pH changes and could resist the influence of acid and alkali on anthocyanin. It was also found that anthocyanin in fibers could be slowly dissolved and dispersed into the buffer solution over time, mainly owing to the water absorption function and swelling property of the CA@Anthocyanin UFs. Fluorescence spectra of the CA@Anthocyanin UFs in different pH buffers were collected over time (Figure S8), and these suggest that the fluorescence signal was without obvious changes while in these buffers, demonstrating that there is a similar dissolution process for anthocyanin from the CA@Anthocyanin UFs. Moreover, there were similar release trends for the CA@Anthocyanin UFs with and without ultraviolet (UV) irradiation (Figure S9). Therefore, the CA@Anthocyanin UFs could stabilize the loaded anthocyanin under different external conditions.

### 3.9. Determination of Controlled Release Properties of CA@Anthocyanin UFs In Vitro

**Figure 8:**
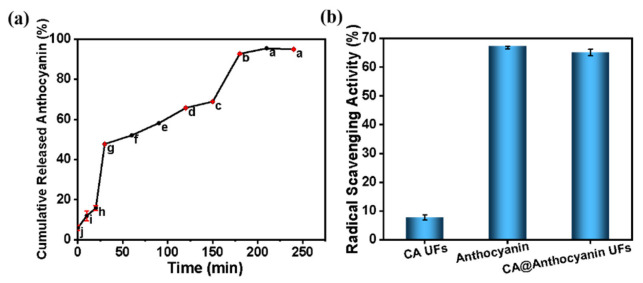
(**a**) Anthocyanin release profile from the CA@Anthocyanin UFs into the artificially simulated gastric fluid; (**b**) the DPPH radical-scavenging activities of CA UFs, the anthocyanin, and CA@Anthocyanin UFs. Note: Different letters (a–j) represent significant differences (*p* < 0.05) between groups.

The artificially simulated gastric fluid with pH = 2.0 was used as a gastric environment to calculate the release rate of anthocyanin loaded on the CA@Anthocyanin UFs in gastric fluid. The release profile of the anthocyanin from the CA@Anthocyanin UFs in the artificially simulated gastric fluid in vitro is shown in Figure 8a. It was observed that there was a fast release rate of anthocyanin in the early stage (within 30 min). At 30 min and 240 min, the release rate of anthocyanin reached 48% and 95%, respectively. At the beginning of the release of anthocyanin, the CA@Anthocyanin UFs had a rapid release period, and the whole release process initially followed a fast rate and then a slow rate. This might be because the anthocyanin near the surface of the fiber had low diffusion resistance and easily diffused into the release solution, resulting in a faster release rate in the early stage. However, the anthocyanin within the superfine fiber needed to reach the surface of the fiber through diffusion and then be released, resulting in a slower release rate in the later stage, which showed that the CA@Anthocyanin UFs could control the release of anthocyanin. These results indicate that the robust electrospun CA UFs have a promising potential with respect to bioactive substance delivery and controlled release.

### 3.10. Antioxidant Activity of Anthocyanin and CA@Anthocyanin UFs

The antioxidant activity of the CA@Anthocyanin UFs was evaluated by the DPPH radical-scavenging assay. In Figure 8b, the CA UFs show weak antioxidant activity, while the average DPPH radical-scavenging rates of anthocyanin and the CA@Anthocyanin UFs were 66.86% and 65.11%, respectively. The results indicate that the antioxidant activity of the CA@Anthocyanin UFs decreased slightly compared with that of anthocyanin, indicating that the CA@Anthocyanin UFs had a weak effect on the antioxidant activity of anthocyanin. However, anthocyanin was loaded by CA, which still had excellent antioxidant activities. These antioxidant activities were mainly attributed to the anthocyanin released from the CA@Anthocyanin UFs, which could capture DPPH radicals [42].

## 4. Conclusions

In summary, cellulose acetate@anthocyanin ultrafine fibers (CA@Anthocyanin UFs) were successfully prepared by employing electrospinning technology, and the loading efficiency, stability, release performance in vitro, and antioxidant properties of the CA@Anthocyanin UFs were further explored. The experimental data showed that the loading efficiency of the CA@Anthocyanin UFs with an anthocyanin content of 7 wt.% reached the optimum levels. The introduction of anthocyanin had no obvious impact on the thermal properties of the cellulose acetate ultrafine fibers (CA UFs). The CA@Anthocyanin UFs exhibited a lower crystallinity, lower mechanical properties, and a higher swelling degree compared with the CA UFs. Furthermore, under the same treatment conditions (temperature and light), the loss rate of anthocyanin from the CA@Anthocyanin UFs was less than that of pure anthocyanin, indicating that the CA@Anthocyanin UFs could stabilize and protect the loaded anthocyanin against different external conditions. The complete release cycle of the CA@Anthocyanin UFs in the artificial gastric fluid was 240 min, which showed its good controlled release performance. The radical-scavenging assay suggested that the CA@Anthocyanin UFs still maintained excellent antioxidant properties. The preparatory process of the CA@Anthocyanin UFs was simple and efficient, and the complex showed good environmental stability, a long release duration, and good antioxidant activity. Thus, the bioactivity and bioavailability of anthocyanin could be improved by the robust electrospun CA UFs, which also showed that robust electrospun CA UFs exhibited a promising potential as a new bioactive nanomaterial and green packaging material with good development prospects.

## Data Availability

All data used for evaluation in this manuscript are herein presented.

## Supplementary Materials

The following supporting information can be downloaded at: https://www.mdpi.com/article/10.3390/polym14194036/s1, Figure S1: Standard curve of anthocyanin aqueous solution with various concentrations; Figure S2: (a–f) SEM images of CA@Anthocyanin UFs with various concentrations of anthocyanin (a—0%, b—3%, c—5%, d—7%, e—9%, and f—11%). Inset: diameter distribution of CA@Anthocyanin UFs; a—0%, b—3%, c—5%, d—7%, e—9%, and f—11%; Figure S3: Captured photo of the CA@Anthocyanin UFs under room light (7 wt.%); Figure S4: TGA curve of anthocyanin; Figure S5: DSC curves of (a) CA UFs and (b) CA@Anthocyanin UFs; Figure S6: XRD patterns of anthocyanin, CA UFs, and CA@Anthocyanin UFs; Figure S7: (a) Typical stress–strain curves; (b) tensile strength; (c) elongation at break of CA UFs and CA@Anthocyanin UFs; Figure S8: Fluorescence spectra of CA@Anthocyanin UFs in the citric acid/sodium citrate buffer solution with different pH values gathered with time interval of 10 min (0–150 min); Figure S9: (a) and (b) Fluorescence spectra of CA@Anthocyanin UFs in water with/without UV irradiation (10, 20, 30, 60, 90, 120, and 150 min), respectively; (c) comparison of the fluorescence intensity of CA@Anthocyanin UFs with/without UV irradiation at different times (10, 20, 30, 60, 90, 120, and 150 min).
