# Supplementary material for: Robust Electrospinning-Constructed Cellulose Acetate@Anthocyanin Ultrafine Fibers: Synthesis, Characterization, and Controlled Release Properties
Source: Polymers (Basel). 2022 Sep 27;14(19):4036. doi: 10.3390/polym14194036 (PMC9571753; doi:10.3390/polym14194036)
Supplement: Supplementary file 1 [file polymers-14-04036-s001.zip › polymers-1892186-supplementary.pdf]

polymers MDPI

Supplementary Materials

Robust Electrospinning-Constructed Cellulose Acetate@Anthocyanin Ultrafine Fibers: Synthesis, Characterization, and Controlled Release Properties

Mingzhu Liu[1,2], Shilei Zhang [2], Yuanyuan Ye [1,2], Xiaoqing Liu [1,2], Jiangling He [1,*], Lingfeng Wei [1,2], Die Zhang [2], Jiaojiao Zhou [1] and Jie Cai [2,*]

[1] National R&D Center for Se-rich Agricultural Products Processing, Hubei Engineering Research Center for Deep Processing of Green Se-rich Agricultural Products, School of Modern Industry for Selenium Science and Engineering, Wuhan Polytechnic University, Wuhan 430023, China
[2] Key Laboratory for Deep Processing of Major Grain and Oil, Ministry of Education, Hubei Key Laboratory for Processing and Transformation of Agricultural Products, Wuhan Polytechnic University, Wuhan 430023, China
* Correspondence: hejiangling@whpu.edu.cn (J.H.); caijievip@whpu.edu.cn (J.C.)

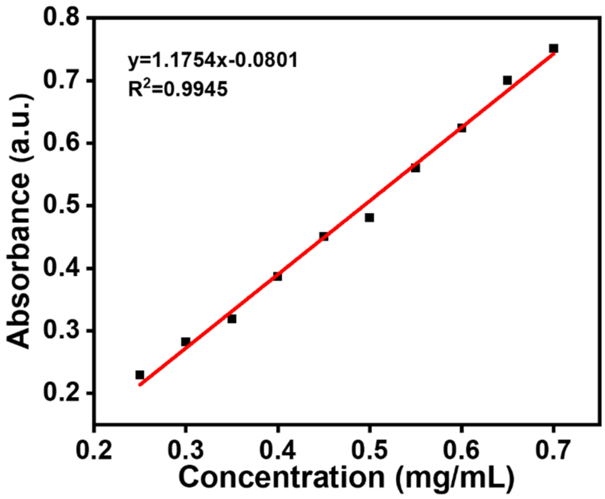

Figure S1. Standard curve of anthocyanin aqueous solution with various concentrations.

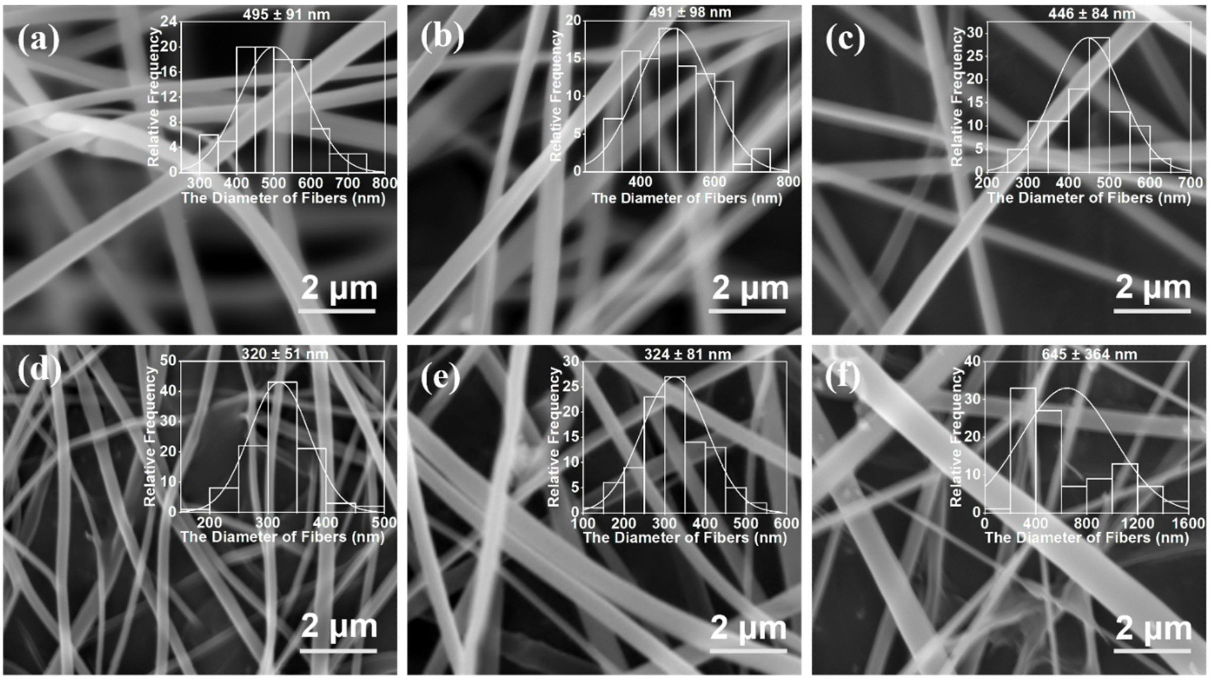

Figure S2. (**a**–**f**) SEM images of CA@Anthocyanin UFs with various concentrations of anthocyanin (**a**-0%, **b**-3%, **c**-5%, **d**-7%, **e**-9%, **f**-11%). Inset: diameter distribution of CA@Anthocyanin UFs; **a**-0%, **b**-3%, **c**-5%, **d**-7%, **e**-9%, **f**-11%.

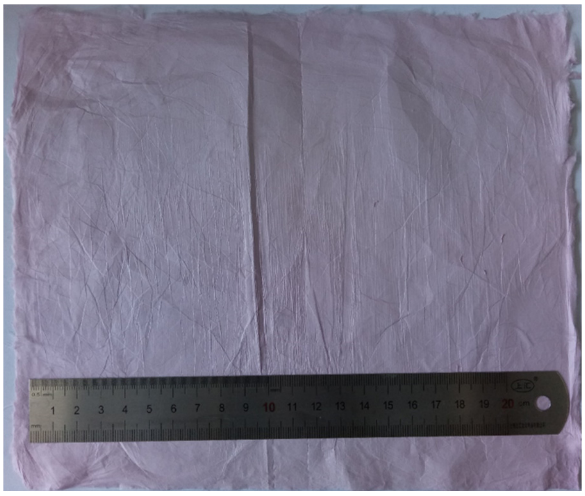

Figure S3. The captured photo of the CA@Anthocyanin UFs under room light (7 wt.%).

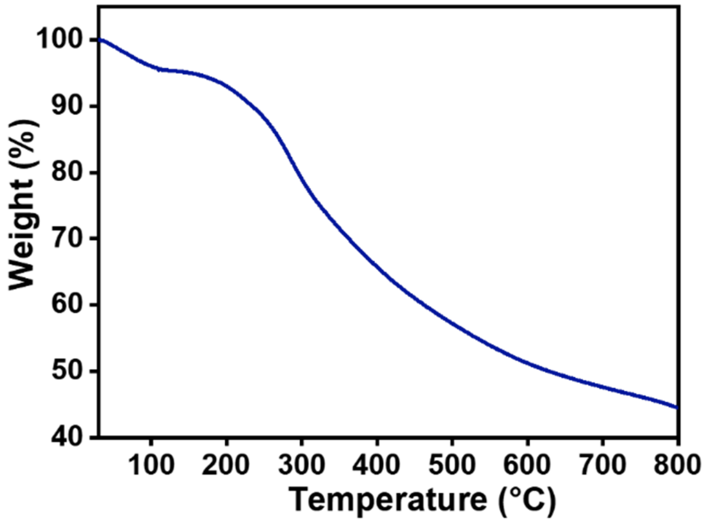

Figure S4. TGA curve of anthocyanin.

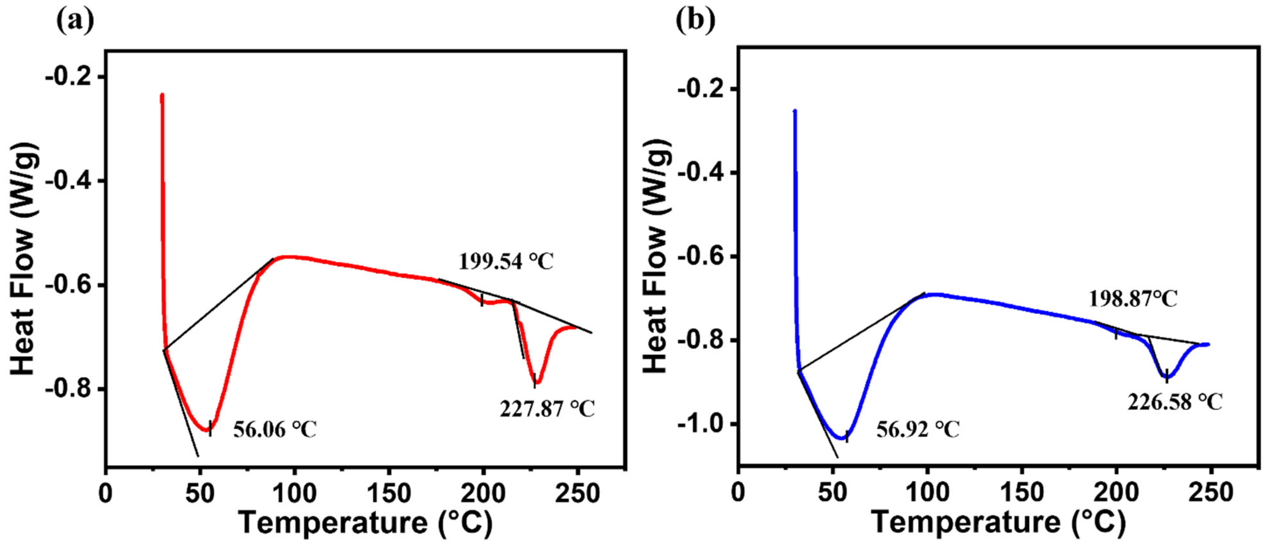

Figure S5. DSC curves of (**a**) CA UFs and (**b**) CA@Anthocyanin UFs.

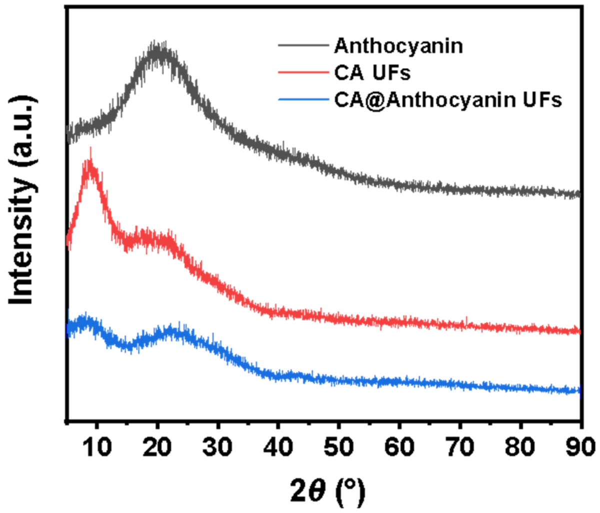

Figure S6. XRD patterns of anthocyanin, CA UFs, and CA@Anthocyanin UFs.

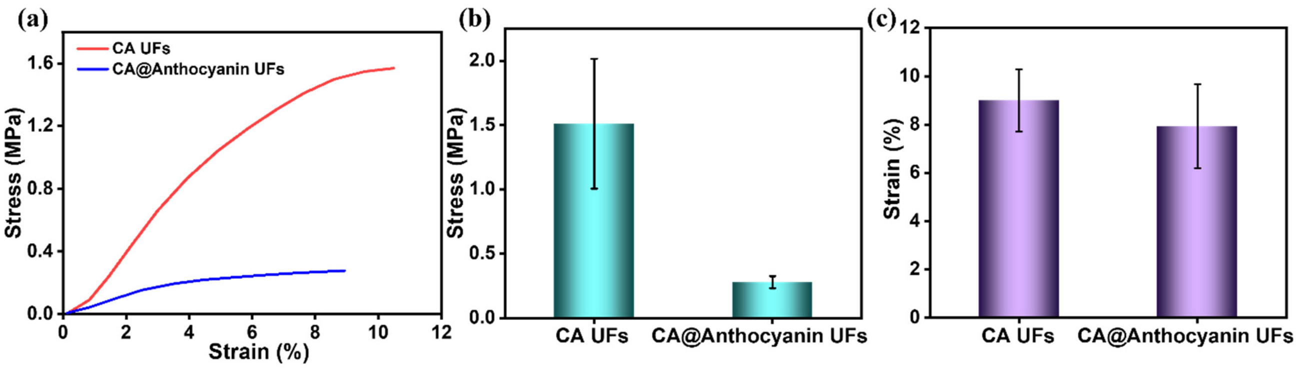

Figure S7. (**a**) Typical stress-strain curves; (**b**) tensile strength; (**c**) elongation at break of CA UFs and CA@Anthocyanin UFs.

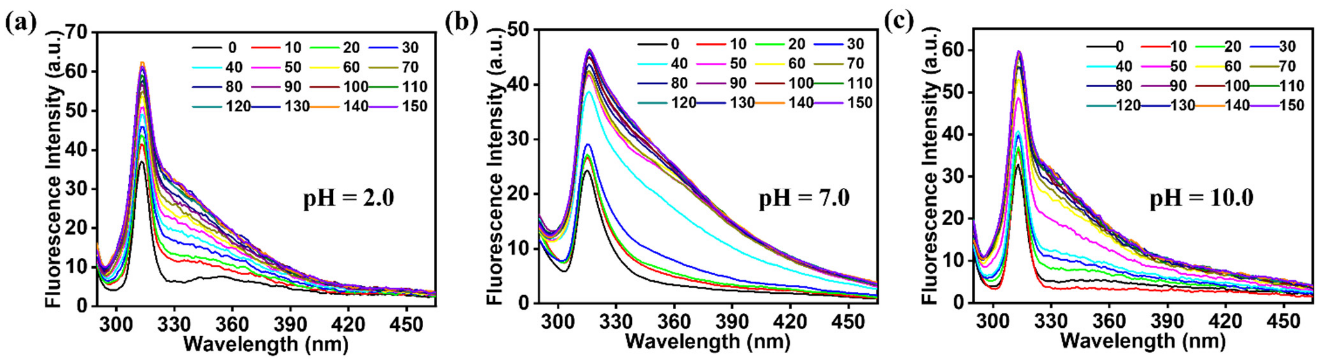

Figure S8. Fluorescence spectra of CA@Anthocyanin UFs in the citric acid/sodium citrate buffer solution with different pH values gathered with time interval of 10 min (0-150 min).

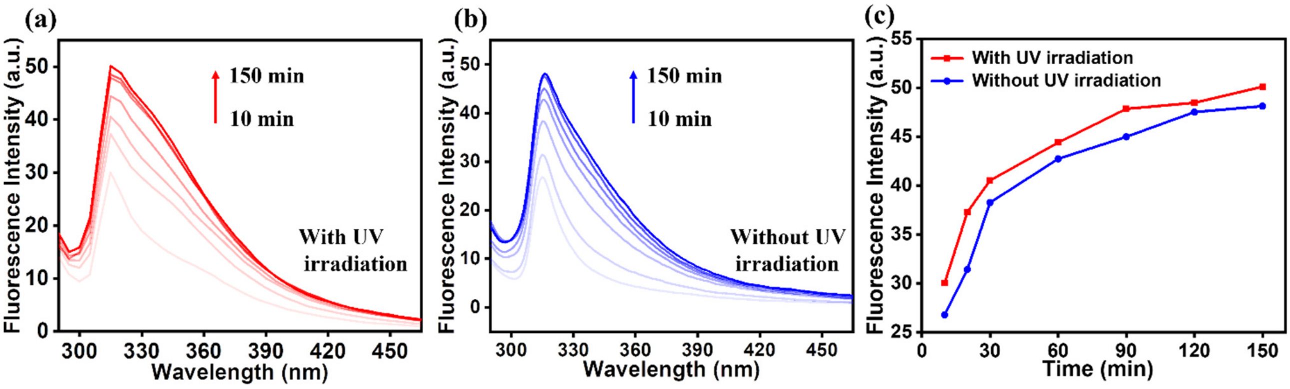

Figure S9. (**a**) and (**b**) Fluorescence spectra of CA@Anthocyanin UFs in water with/without UV irradiation (10, 20, 30, 60, 90, 120, and 150 min), respectively; (**c**) comparison of the fluorescence intensity of CA@Anthocyanin UFs with/without UV irradiation at different times (10, 20, 30, 60, 90, 120, and 150 min).
